# Focused analysis of the surgical management for recurrence of internal rectal prolapse and obstructed defecation syndrome after stapled rectal resection procedures: a long-term single-center experience

**DOI:** 10.1007/s00384-026-05167-x

**Published:** 2026-06-11

**Authors:** Angelo A. Marra, Claudia Varrella, Cesare Caruso, Ilaria Simonelli, Angelo Parello, Francesco Litta, Paola Campennì, Mario Pagano, Carlo Ratto

**Affiliations:** 1 Proctology and Pelvic Floor Surgery Unit, Center of Excellence for Gastrointestinal and Endocrine-Metabolic Diseases, Isola Tiberina - Gemelli Isola Hospital, Via Di Ponte Quattro Capi, 39, Rome, 00186 Italy; 2 Biostatistical Service, Clinical Trial Center, Isola Tiberina - Gemelli Isola Hospital, Rome, Italy; 3 Department of Biomedicine and Prevention, University of Rome Tor Vergata, Rome, Italy; 4 Department of Medical and Surgical Sciences, Università Cattolica del Sacro Cuore, Rome, Italy

**Keywords:** Internal rectal prolapse, Obstructed defecation syndrome, Stapled rectal resection, Rectal prolapse surgery, Recurrence surgery

## Abstract

**Background:**

Stapled rectal resection procedures have been adopted for the treatment of internal rectal prolapse (IRP) and obstructed defecation syndrome (ODS). However, concerns remain in case of surgery for recurrence. This study aimed to analyze the impact of a staple-line scar in patients undergoing surgery for IRP and ODS recurrence.

**Methods:**

A prospective maintained database of patients who underwent abdominal or perineal surgery for IRP and ODS between November 1998 and January 2025 was retrospectively analyzed. Patients with a history of stapled rectal resection procedures were specifically evaluated. Baseline clinical and radiological characteristics, surgical complexity related to stapled suture, postoperative complications, recurrence, and ODS and fecal incontinence scores were collected.

**Results:**

Of 376 female patients, 50 (13.3%) with prior stapled rectal resection procedures underwent surgery for IRP and ODS recurrence. In three cases, the staple-line scar could not be safely overcome. At last follow-up, recurrence and complication rates were comparable between patients with and without previous stapled procedures (4.8% vs. 6.1% and 19.0% vs. 18.4%, respectively). One patient in the stapled group experienced persistent pelvic pain despite anatomical correction. Although ODS and fecal incontinence scores improved overall, patients with prior stapled rectal resection reported higher ODS scores. However, linear mixed-effects analysis did not demonstrate statistically significant differences between groups.

**Conclusions:**

Surgery for IRP and ODS recurrence after stapled rectal resection procedures is challenging. Although previous stapled rectal resection procedures may be associated with higher ODS scores, further studies are needed to clarify these findings.

**Supplementary information:**

The online version contains supplementary material available at 10.1007/s00384-026-05167-x.

## Introduction

The Stapled Trans-Anal Rectal Resection (STARR) procedure, an evolution of the Procedure for Prolapse and Hemorrhoids (PPH) technique [1, 2], was developed as a minimally invasive transanal procedure aimed at correcting not only hemorrhoidal prolapse but also anatomical rectal abnormalities, such as internal rectal prolapse (IRP), and symptoms of obstructed defecation syndrome (ODS) [3]. Specifically, it involves a double hemi-circumferential transanal resection of the rectal wall using a surgical stapling device, designed to remove more rectal tissue than the PPH technique, thereby restoring anorectal anatomy and improving evacuation. Over time, several modifications have been described in the literature [4, 5], including the trans-STARR procedure, which was developed to enable more extensive, tailored circumferential correction of the IRP under continuous visual control using a Contour stapler [6].

Initially, all types of stapled rectal resection procedures were welcomed with considerable enthusiasm because of their feasibility and favorable short-term outcomes, including reduced pain, shorter hospital stays, and a quicker return to work [3, 7, 8]. However, subsequent evidence increasingly raised concerns regarding their safety profile, long-term efficacy, and the risk of serious complications, including bleeding, fecal urgency and incontinence, persistent anorectal pain, stenosis, rectovaginal fistula, pelvic sepsis, and the need for reoperation [8–14]. As a result, several alternative surgical techniques, including abdominal approaches, have been explored over time for the management of patients with IRP and ODS. Among these, ventral mesh rectopexy (VMR) has gained increasing clinical and scientific interest because of its ability to simultaneously correct additional anatomical abnormalities of the posterior compartment, such as enterocele, as well as disorders of the anterior and middle compartments when combined with other surgical procedures [15–17].

However, when other surgical techniques are performed after any type of stapled rectal resection procedure, the operation is often considered more technically challenging and potentially riskier [18]. Furthermore, the effect of previous stapled rectal resection procedures in patients with IRP and ODS recurrence has rarely been evaluated [18–20]. Therefore, the aim of this study was to analyze the impact of a fixed staple-line scar following stapled rectal resection procedures in patients undergoing surgery for recurrence of IRP and ODS within a long-term follow-up database.

## Methods

### Study design

A retrospective cohort analysis of a prospectively maintained single-center database was conducted on patients who underwent abdominal and perineal surgery for IRP, with or without rectocele and entero-/sigmoidocele, and ODS symptoms at our tertiary academic center from November 1998 to January 2025. Patients who had previously undergone stapled rectal resection procedures at other centers and presented with IRP and ODS recurrence were specifically evaluated. In this study, exclusion criteria included ERP, malignancies under treatment, inflammatory bowel disease, chronic diarrhea unresponsive to medical treatment, pregnancy, and inability to provide written informed consent. The study was approved by the Ethical Committee of our institution (ID 6120), and written informed consent was obtained from each patient. The Strengthening the Reporting of Observational Studies in Epidemiology (STROBE) statement for cohort studies was followed [21].

### Data assessment

Overall, patients’ baseline characteristics were systematically recorded, including demographic data, comorbidities (described using the ASA classification), preoperative anxiety and depression, previous abdominal and perineal surgery (including hysterectomy), pelvic trauma or radiotherapy, vaginal delivery (including episiotomy, obstetric anal sphincter injury, forceps or vacuum use), cesarean section, preoperative pelvic floor rehabilitation, ODS, and fecal (FI) and urinary incontinence symptoms. Clinical and radiological findings were also collected, including fluoroscopic or MRI defecography, colonic transit time, endoanal ultrasound, anorectal manometry, and colonoscopy when performed. Over time, several abdominal and perineal techniques were adopted for the treatment of IRP and ODS, depending on the surgeon’s experience and preference. Among the abdominal procedures (performed via open or minimally invasive approaches, including robotic surgery) were as follows: VMR, in which the mesh is fixed distally to the ventral aspect of the rectum (after anterior rectovaginal dissection) and proximally to the sacral promontory; the Orr-Loygue procedure, involving extensive posterior rectal mobilization with distal mesh fixation to the ventral rectum and proximal anchoring to the sacral promontory; and the Frykman-Goldberg procedure, consisting of sigmoidectomy with suture rectopexy at the sacral promontory. During VMR, two radiopaque clips were placed at the most distal edge of the mesh on the ventral rectal wall, two clips at the level of the mesh’s proximal fixation over the rectum, two additional clips on the mesh at the sacral promontory, one further clip on the sacral periosteum, and another on the perineal body. This technical modification was introduced to enable subsequent radiographic assessment of the mesh position during follow-up, especially in cases of suspected mesh detachment. Conversely, the perineal procedures included the internal Delorme’s technique, involving mucosectomy of the redundant rectal mucosa and plication of the muscular layer, and transvaginal rectocele repair, in which horizontal plication sutures reduce rectal protrusion into the rectovaginal septum. No stapled rectal resection procedures were performed at our center. All surgical procedures were performed by the same colorectal surgeon (C.R.).

Among patients who underwent surgery for IRP and ODS recurrence after stapled rectal resection procedures, surgical complexity related to the stapled suture was documented. Postoperative complications and recurrence of IRP were recorded at the last follow-up visit. Validated patient-reported outcome measures (PROMs) were collected preoperatively and at 1, 6, and 12 months postoperatively, and then annually during follow-up visits. Specifically, ODS severity was assessed with the Altomare ODS score [22], and FI symptoms were evaluated using the Cleveland Clinic Fecal Incontinence (CCFI) score [23]. Among PROMs for ODS symptoms, the Altomare ODS score was selected because it could be reliably reconstructed in patients treated before its introduction using individual items that had already been systematically recorded, particularly in terms of symptom frequency.

### Statistical analysis

Categorical data were reported as frequencies and percentages, and continuous variables as medians and interquartile ranges (IQR, 25th–75th percentiles). The chi-square test was used to assess associations between categorical variables. Given non-normal distributions, differences in continuous variables between the two groups were analyzed using the Mann-Whitney *U* test. Pre- and postoperative differences in continuous variables were evaluated using the Wilcoxon signed-rank test. A linear mixed-effects model was used to evaluate pre- and postoperative changes in the Altomare ODS score between the previous and non-stapled rectal resection groups, adjusting for variables that differed between groups in the univariate analysis. A random intercept for each subject was included to account for within-subject correlation. Patients with any missing data were excluded from the analysis. A *p *value < 0.05 was considered statistically significant. Statistical analysis was performed using IBM SPSS Statistics for Windows, version 26.0 (IBM Corp, Armonk, NY, USA).

## Results

**Fig. 1:**
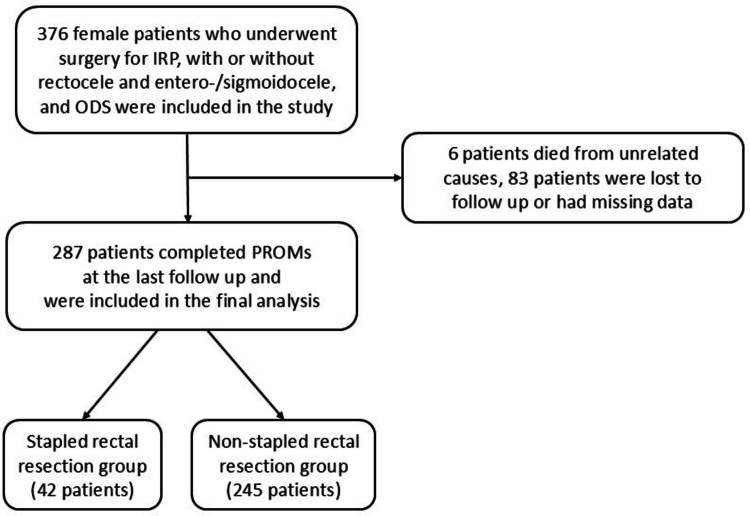
Flow diagram illustrating the selection and inclusion criteria for patients in the statistical analysis. IRP = internal rectal prolapse; ODS = obstructed defecation Syndrome; PROMs = patient-reported outcome measures

During the study period, 50 out of 376 (13.3%) female patients who underwent surgery for IRP, with or without associated rectocele and entero-/sigmoidocele, and ODS had a history of stapled rectal resection procedures (40 STARR-like, 8 PPH-like, and, notably, 2 involving both techniques) and were included in the stapled rectal resection group. The study flowchart is shown in Fig. 1.

### Preoperative clinical and radiological assessments

**Fig. 2:**
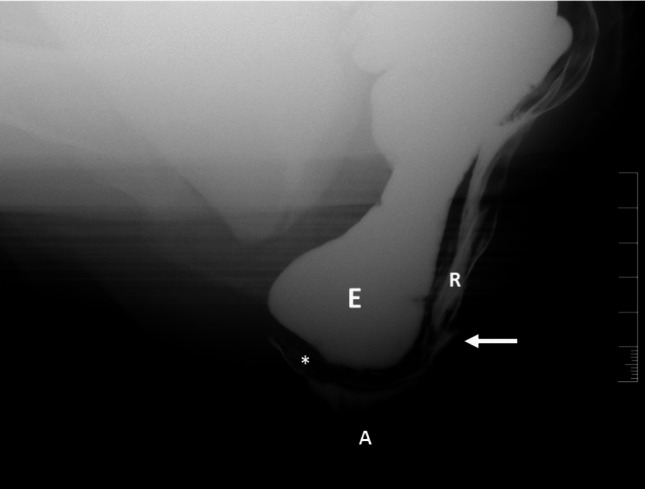
Defecographic snapshot showing a large enterocele (E) compressing the rectum (R) and the rectocele (*), which hinders their emptying. An intra-anal intussusception (indicated by the white arrow, likely at the level of the stapled rectal resection suture) is also present. E = enterocele; R = rectum; A = anal canal; * = rectocele

Baseline characteristics are summarized in Table 1. Preoperative fluoroscopic or MRI defecography data are presented in Table 2. In the stapled rectal resection group, IRP recurrence was detected in 42 (85.7%) patients, with 14 (28.6%) exhibiting intrarectal and 28 (57.1%) exhibiting intra-anal intussusception. Similarly, rectocele was observed in 42 of 50 (85.7%) cases, with a median size of 40.0 (IQR: 28.0–50.0) mm. Additionally, residual mucosal prolapse was detected in 21 (42.0%) patients who had undergone previous stapled rectal resection surgery, whereas entero-/sigmoidocele was found in 33 (66.0%) cases on fluoroscopic defecography (Fig. 2). Compared with the rest of the cohort, the stapled rectal resection group had a higher prevalence of mucosal prolapse (previous stapled rectal resection procedure group: 21 cases, 42.9%; chi-square test *p* = 0.008), whereas rectocele was more commonly detected in the non-stapled rectal resection group, with greater dimensions [non-stapled rectal resection procedure group: 45.0 (38.0–50.0) mm; Mann-Whitney test *p* = 0.012]. No significant differences were observed for the other variables.

**Table 1:** Baseline characteristics and clinical data of patients with or without previous stapled rectal resection procedure who underwent surgery for internal rectal prolapse and obstructed defecation syndrome

Baseline characteristics	Previous stapled rectal resection group (50 females)	Non-stapled rectal resection group (326 females)	*p* value
Age, years*	55.5 (49.0–68.3)	58.0 (51.0–66.0)	0.570^a^
Age < 40 years	5 (10.0%)	19 (5.9%)	0.267
ASA 3	9 (18.0%)	30 (9.3%)	0.107
Anxiety/depression	20 (40.0%)	91 (28.1%)	0.204
Hysterectomy	14 (28.0%)	93 (28.7%)	0.918
Pelvic trauma/radiotherapy	2 (4.0%)	16 (4.9%)	0.773
Vaginal deliveries	31 (62.0%)	248 (76.5%)	**0.028**
No. vaginal deliveries*	1 (0–2)	2 (1–2)	0.182^a^
OASIs	9 (18.0%)	83 (25.6%)	0.249
Episiotomy	21 (42.0%)	151 (46.6%)	0.559
Forceps/vacuum	4 (8.0%)	22 (6.8%)	0.747
Cesarean section	9 (18.0%)	68 (21.0%)	0.637
Preoperative PF rehabilitation	13 (26.0%)	46 (14.2%)	0.073
Slow colonic transit	10 (20.0%)	39 (11.7%)	0.181

*Data are shown as median (interquartile range, IQR). *p *values are referred to the chi-square test, statistically significant *p*-value are shown in bold. ^a^The *p *value is referred to as the non-parametric Mann-Whitney test, statistically significant *p*-value are shown in bold. *ASA* American Society of Anesthesiologists, *OASIs* obstetric anal sphincter injuries, *PF* pelvic floor

**Table 2:** Data regarding preoperative fluoroscopic or MRI defecography of patients with or without previous stapled rectal resection procedure who underwent surgery for internal rectal prolapse and obstructed defecation syndrome

Preoperative defecographic data	Previous stapled rectal resection group (50 females)	Non-stapled rectal resection group (326 females)	*p* value
Patients	49 (98.0)	309 (94.8)	0.321
MRI defecography	8 (16.3)	32 (10.4)	0.412
Incomplete rectal empty	23 (46.9)	153 (49.5)	0.722
Dyssynergia	11 (22.4)	52 (16.8)	0.342
Descent perineum (no. pts)	31 (63.3)	197 (63.8)	0.925
Descent perineum (mm)*	60.0 (50.0–65.0)	55.0 (50.0–60.0)	0.456^a^
Rectocele (no. pts.)	42 (85.7)	293 (94.8)	**0.037**
Rectocele (mm)^*^	40.0 (28.0–50.0)	45.0 (38.0–50.0)	**0.012**^**a**^
Rectocele empty	5 (10.2)	19 (6.1)	0.296
Intra-rectal intussusception	14 (28.6)	126 (40.8)	0.104
Intra-anal intussusception	28 (57.1)	139 (45.0)	0.113
Mucosal prolapse	21 (42.9)	76 (24.6)	**0.008**
Entero-/sigmoido-/peritoneocele	33 (67.3)	176 (57.0)	0.122
Colpo-hysterocele	21 (42.9)	133 (43.0)	0.937
Cystocele	23 (46.9)	139 (45.0)	0.864

*Data are shown as median (interquartile range, IQR). *p *values are referred to the chi-square test, statistically significant *p*-value are shown in bold. ^a^The *p *value is referred to as the non-parametric Mann-Whitney test, statistically significant *p*-value are shown in bold. *MRI* magnetic resonance imaging

### Surgical details and postoperative outcomes

**Fig. 3:**
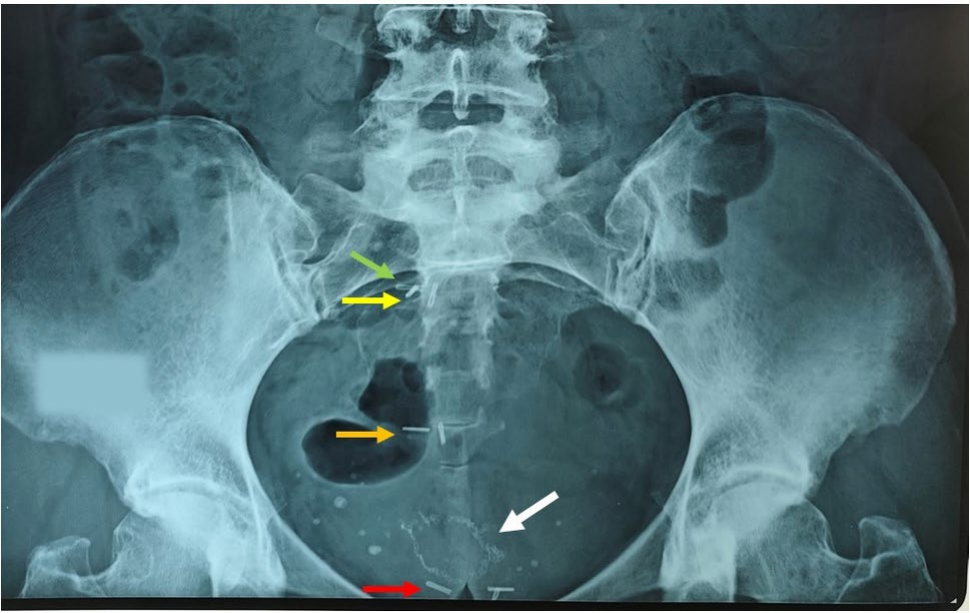
During ventral mesh rectopexy, the stapled rectal resection suture (indicated by the white arrow) was successfully overcome during rectovaginal dissection, as documented by the postoperative X-ray assessment of radiopaque clips. They were intraoperatively positioned at the level of the sacral promontory (1 clip indicated by the green arrow) and on the mesh (2 clips at the sacral promontory level—yellow arrow, 2 clips at the level of the most proximal pair of sutures on the ventral rectum—orange arrow, and 2 clips at the level of the most distal pair of sutures on the ventral rectum—red arrow, beyond the stapled suture) to allow mesh assessment during the follow-up

**Fig. 4:**
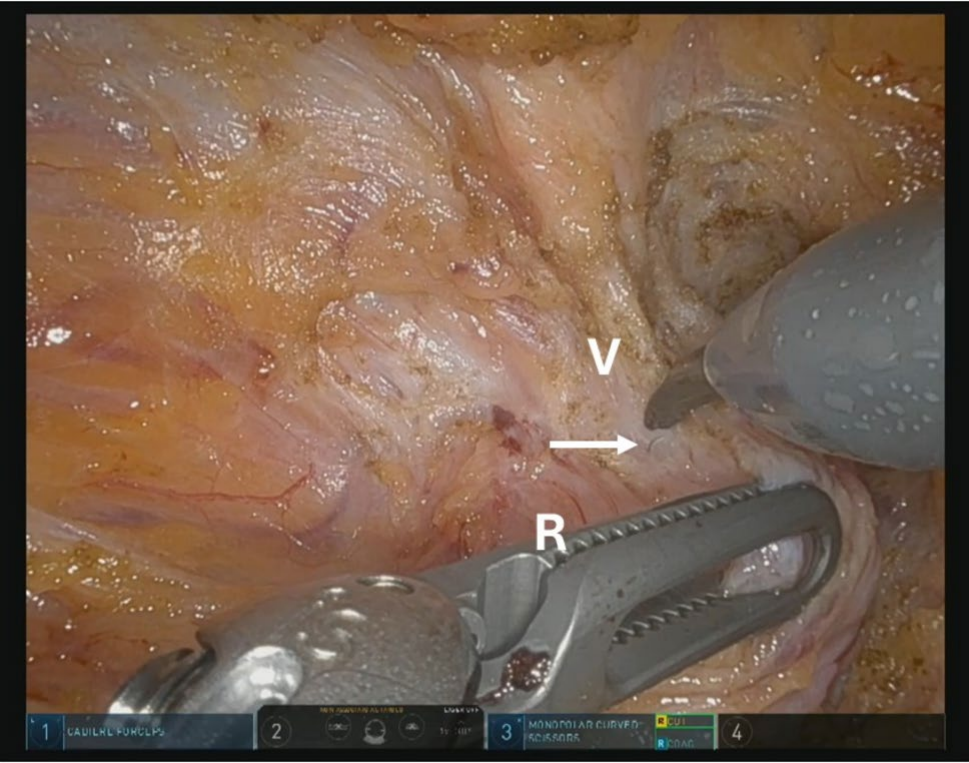
During robotic ventral mesh rectopexy, the stapled rectal resection suture line increases the technical complexity of the rectovaginal dissection and the risk of rectal and vaginal injury. In this intraoperative image, a clip of the stapled suture (indicated by the white arrow) can be observed beyond the rectal serosa during the rectovaginal dissection. R = ventral rectum; V = posterior vaginal wall

Considering all patients, 265 VMR procedures were performed, along with 26 Orr-Loygue and 4 Frykman-Goldberg approaches among the abdominal procedures. Perineal approaches included 68 transvaginal rectocele repairs and 13 internal Delorme’s procedures over time. More specifically, in the stapled rectal resection group, 45 patients underwent an abdominal procedure (41 VMR, 2 Orr-Loygue, and 2 Frykman-Goldberg procedures), while 5 patients underwent a perineal approach (4 transvaginal rectocele repairs and 1 internal Delorme’s procedure). Although in most cases the stapled suture was successfully overcome during rectovaginal dissection (as documented by postoperative X-ray, Fig. 3), this was not possible in three patients (two undergoing VMR and one undergoing transvaginal rectocele repair) due to technical complexity and the increased risk of rectovaginal injury, as in all three cases the fixed scar related to the stapled line was located at the level of the residual rectocele. Consequently, in the two abdominal procedures (one of which was performed using a robotic approach, Fig. 4), it was not possible to dissect the rectovaginal septum or to position the mesh on the ventral rectal wall as distally as intended. Likewise, in the perineal procedure, the repair of the residual rectocele could only be performed below the suture line.

Overall, 287 of 376 (76.3%) patients completed the questionnaires at the last follow-up visit, including 42 in the stapled rectal resection group and 245 in the non-stapled rectal resection group (Fig. 1). Six patients in the non-stapled rectal resection group had died from unrelated causes. The median follow-up was 53.0 (IQR: 14.0–115.0) months; there was no significant difference between the two groups [53.0 (IQR: 21.3–90.3) versus 53.0 (IQR: 13.8–123.3) months in the previous versus non-stapled rectal resection group; Mann-Whitney test *p* = 0.502]. A total of 17 (5.9%) recurrences of IRP were documented by postoperative clinical and defecographic evaluation, with similar rates between the previous and non-stapled groups [2 (4.8%) in patients who had previously undergone stapled rectal resection versus 15 (6.1%) in those without a stapled rectal resection, chi-squared test *p* = 0.730]. Of these, 2 and 11 patients in the previous and non-stapled rectal resection groups, respectively, underwent further surgery for recurrent IRP and ODS. Likewise, among the 53 (18.5%) overall postoperative complications, no significant differences were observed between groups [8 (19.0%) in patients who had previously undergone stapled rectal resection procedures versus 45 (18.4%) in those without a stapled rectal resection suture, chi-square test *p* = 0.916]. In particular, in one patient with a history of stapled rectal resection surgery, chronic pelvic pain related to the staple line persisted despite surgery having corrected posterior compartment anatomy.

### Patient-reported outcome measurement

**Fig. 5:**
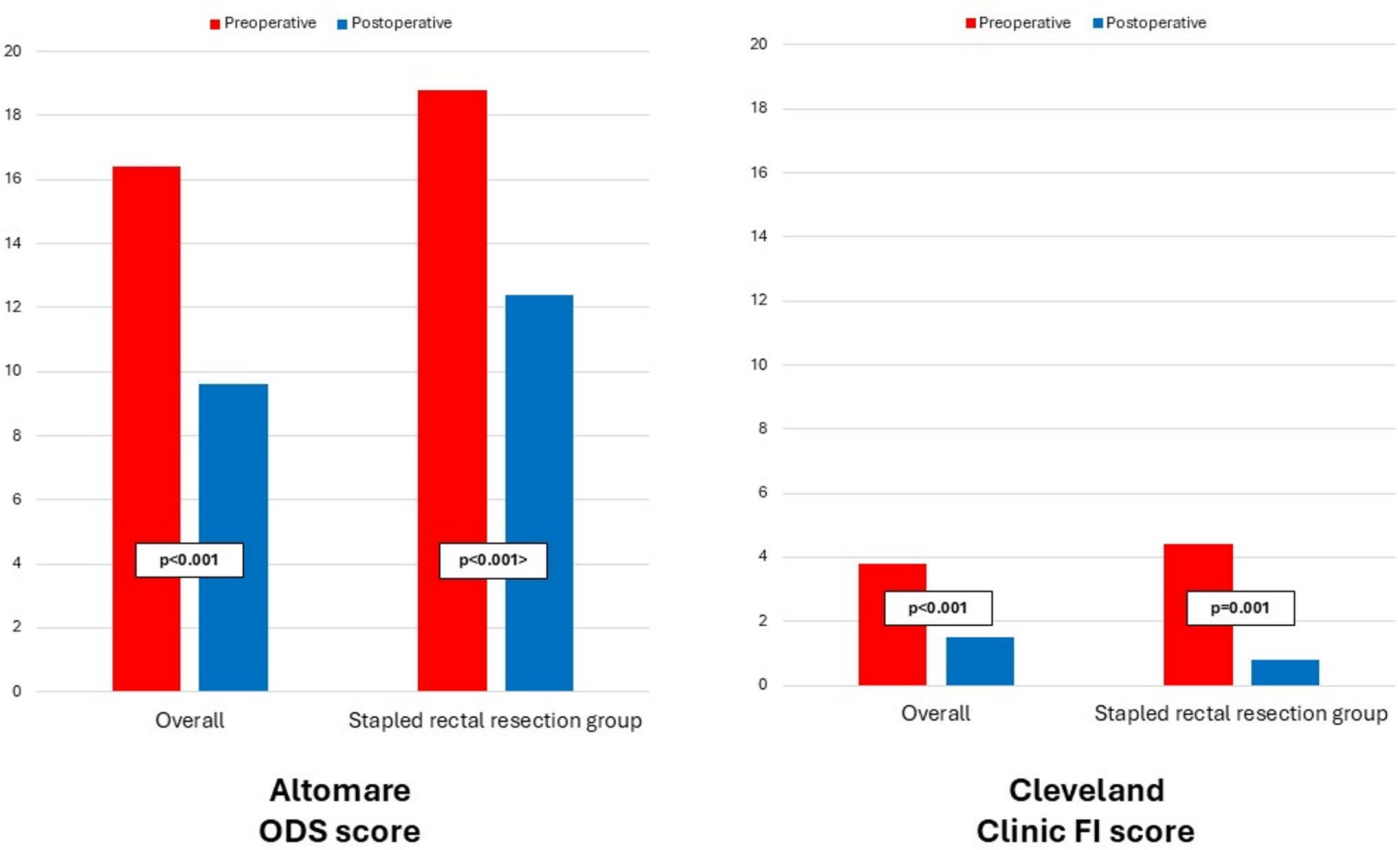
Statistical comparison between preoperative and postoperative Altomare Obstructed Defecation Syndrome and Cleveland Clinic Fecal Incontinence scores in patients who underwent surgery for internal rectal prolapse and obstructed defecation syndrome at our unit, even in patients with a history of stapled rectal resection procedures. *p *values are referred to non-parametric Wilcoxon test. ODS = obstructed defecation syndrome; FI = fecal incontinence

**Fig. 6:**
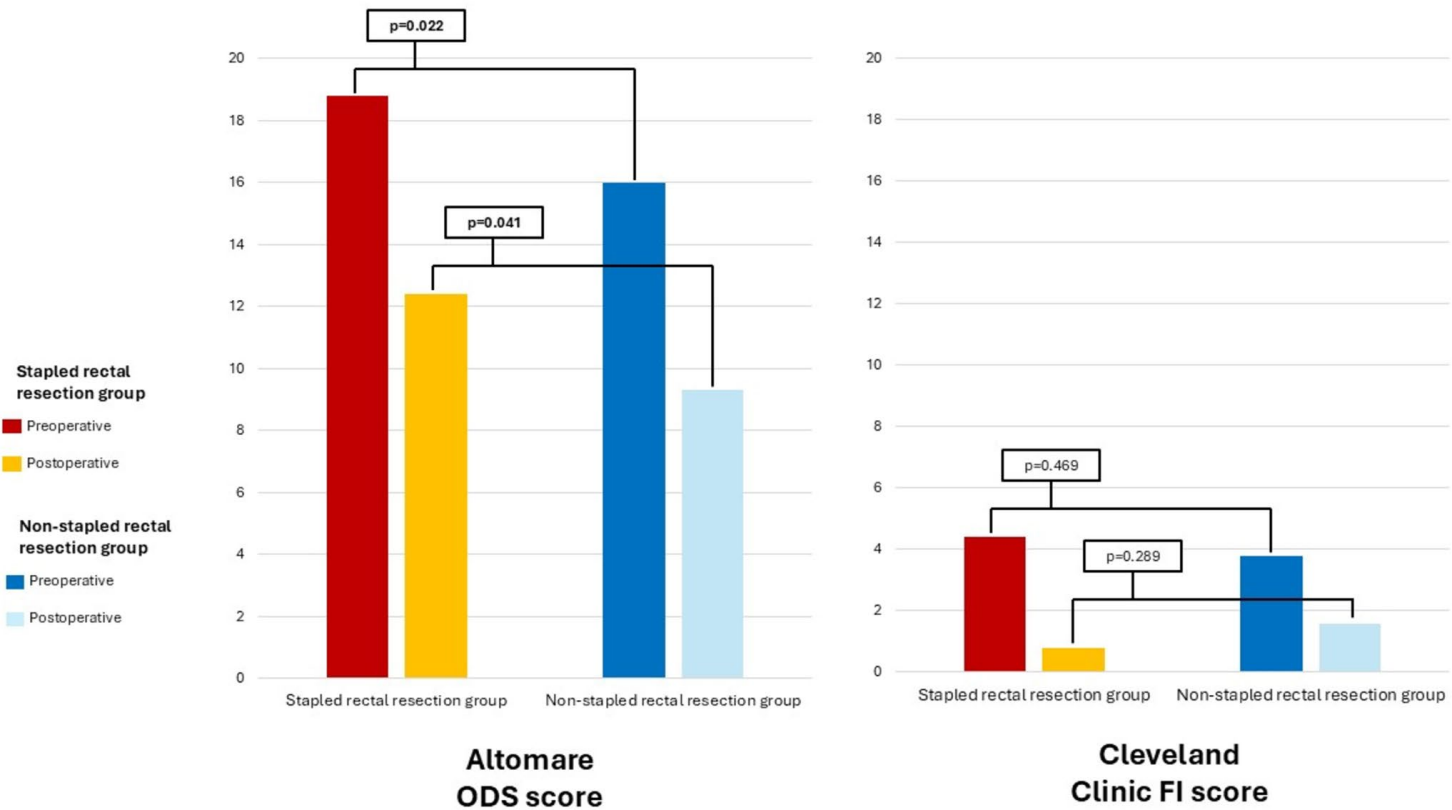
Statistical comparison between Altomare Obstructed Defecation Syndrome and Cleveland Clinic Fecal Incontinence scores in patients with or without previous stapled rectal resection procedure who underwent surgery for internal rectal prolapse, rectocele, and obstructed defecation syndrome. *p *values are referred to as the non-parametric Mann-Whitney test. ODS = obstructed defecation syndrome; FI = fecal incontinence

Overall, Altomare ODS and CCFI scores showed statistically significant reductions after surgery (16.4 ± 6.0 versus 9.6 ± 6.7, and 3.8 ± 5.0 versus 1.5 ± 3.9; Wilcoxon test *p* < 0.001), even among patients with a history of stapled rectal resection surgery (18.3 ± 7.0 versus 12.4 ± 8.0, and 3.3 ± 3.8 versus 0.5 ± 2.4; Wilcoxon test *p* < 0.001 and *p* = 0.001, respectively, Fig. 5). The comparison of PROMs for ODS and FI between previous and non-stapled rectal resection groups is shown in Fig. 6. As shown, patients who had previously undergone any stapled rectal resection procedures had a higher preoperative Altomare ODS score than those without stapled rectal resection suture (18.3 ± 7.0 in the previous stapled rectal resection group versus 16.0 ± 5.8 in the non-stapled rectal resection group; Mann-Whitney test *p* = 0.022). Similarly, postoperatively, the Altomare ODS score remained significantly higher in the stapled rectal resection group (12.4 ± 8.0 versus 9.3 ± 6.5; Mann-Whitney test *p* = 0.041). Linear mixed-effects model analysis showed that, after adjusting for mucosal prolapse and rectocele size, the differences in ODS score between the previous and non-stapled rectal resection group were not significant preoperatively (mean difference = −3.26, 95% CI = −8.24, 1.72; standard error = 1.93; *p* = 0.331), whereas a borderline significant difference was observed postoperatively (mean difference = −4.42, 95% CI = −9.27, 0.43; standard error = 1.88; *p* = 0.088). No differences were observed in overall satisfaction after surgery (6.9 ± 3.3 in the stapled rectal resection group versus 7.3 ± 2.9 in the non-stapled rectal resection group; Mann-Whitney test *p* = 0.629).

## Discussion

The retrospective analysis of our surgical database focusing on patients with prior stapled rectal resection procedures presenting with IRP and ODS recurrence showed that surgery in this setting is still challenging. Initially described as involving only mucosal and submucosal excision, histopathological studies later demonstrated the presence of muscular fibers in more than 90% of surgical specimens, indicating that the resection was far more extensive than originally believed [8, 24, 25]. However, removal of additional rectal tissue (including the muscular layer), as described in more aggressive stapled rectal resection techniques, has not consistently translated into improved postoperative outcomes [10, 26–30]. Conversely, these procedures have been associated with a significant risk of anatomical and functional alterations in the middle and posterior pelvic compartments, including rectal deformity, reduced rectal compliance, and effects consistent with a “full-thickness” excision, all of which are exacerbated by a fixed staple-line scar, which may also preclude further surgical intervention [2, 9, 25, 31]. Indeed, as observed in our case series, even in the hands of surgeons experienced in rectal prolapse surgery, it was not possible to overcome the staple-line scar in 3 out of 50 patients due to the considerable intraoperative risk of vaginal or, even more critically, rectal perforation. Therefore, we suggest exercising caution when surgically managing these patients, ensuring that all risks are clearly discussed during preoperative counseling, and that, whenever possible, surgery is performed in high-volume referral centers with extensive experience in rectal prolapse surgery.

Another interesting aspect emerging from our long-term experience in the surgical management of IRP and ODS is that stapled rectal resection procedures may be associated with recurrence of posterior compartment abnormalities, sometimes related to inappropriate indications, such as the presence of an enterocele [26, 32, 33]. Notably, in our cohort, approximately 66% of patients who had previously undergone stapled rectal resection also presented with an enterocele. This represents a substantial risk during stapled rectal resection surgery, as intestinal loops within the enterocele may become entrapped in the stapled suture line, potentially leading to severe complications such as an entero-vaginal fistula and the consequent need for stoma creation. Preventing strategies such as intraoperative Trendelenburg positioning or laparoscopic abdominal assistance have been proposed to reduce this risk [30, 34]. However, in such cases, we recommend a careful reassessment of surgical indications, considering an abdominal approach, which may provide a more comprehensive and appropriate treatment.

Although the overall benefit of surgery in the treatment of IRP and ODS was also evident in our experience, it was not always substantial and remained burdened by recurrences and complications, regardless of the surgical approach adopted, highlighting the complexity of managing these patients [35, 36]. However, patients who had previously undergone stapled rectal resection procedures appeared to experience greater severity of both preoperative and postoperative ODS symptoms compared with the non-stapled group. Similarly, FI symptoms were not negligible. Likewise, the case of persistent chronic pelvic pain observed in our study could also be related to nerve entrapment in the staple-line scar and was not resolved even after restoration of rectal anatomy.

Nevertheless, the linear mixed-effects model analysis showed that, after adjusting for variables differing between the groups in the univariate analysis (mucosal prolapse and rectocele size), the difference in ODS score was only borderline significant postoperatively. Similarly, recurrence and complication rates in patients who had previously undergone stapled rectal resection surgery were essentially comparable to those observed in patients without a prior staple-line suture. Therefore, it is possible that other factors, often unknown or underestimated, may also influence defecatory mechanics [35, 36]. Unfortunately, a major current limitation in rectal prolapse surgery is the lack of tests that directly assess rectal function in such cases. Although indirect tools such as anorectal and colonic manometry, rectal barostat, rectal scintigraphy, and endorectal capsules have been used to track intestinal movements during defecation [36–38], a comprehensive and objective assessment of rectal function is still lacking. Consequently, the “real” effect of the presence of inert scar tissue, metallic clips, anastomoses between discontinuous layers of muscular fibers, nerve disruption or entrapment within the staple-line suture, and reduced rectal reservoir capacity related to stapled rectal resection procedures on rectal function during the defecation process remains unclear and requires further investigation [19, 20, 26, 39].

This study had several limitations. First, all stapled rectal resection procedures were grouped together because detailed operative information was incomplete or unavailable for some patients. Although different techniques were introduced over time, and some only after the beginning of the study period (e.g., the STARR procedure), the rationale for grouping them was the shared presence of a staple-line scar at the rectal level. Second, as previously noted, it was not possible to determine whether the observed outcomes were attributable to the stapled rectal resection procedure itself or to patient-related factors associated with ODS, as no validated tool is currently available to directly assess rectal function. Likewise, the linear mixed-effects model was not conclusive in clarifying this issue, likely because it was limited to variables showing differences at univariate analysis. Third, despite the long-term nature of our database, the number of patients who had undergone stapled rectal resection procedures was relatively small, and it was not possible to perform separate analyses of abdominal and perineal procedures, although this would have been valuable. However, this reflects real-world clinical practice in managing IRP and ODS recurrence after stapled rectal resection surgery, where an initial conservative approach is generally preferred due to the high risks associated with reoperation in these patients, as previously discussed. Fourth, this study reflects the long-term experience of a single surgeon (C.R.) in rectal prolapse surgery. Since MRI defecography was introduced later than fluoroscopic defecography [40], the diagnostic work-up slightly evolved over time. Similarly, PROMs were progressively adopted, and for patients treated before their introduction, scores were reconstructed using clinical items that had already been systematically collected (see Methods section). Likewise, surgical techniques and surgeon preference also evolved throughout the study period. Therefore, all these aspects should be considered when interpreting the results. Lastly, as this was a retrospective analysis, no formal sample size calculation was performed. In addition, given the long study period, there was a notable loss to follow-up and missing data (23.7%), which may have influenced the final results. Consequently, residual confounding cannot be excluded, and some findings may have been under- or overestimated.

Future comparisons with data from other tertiary referral centers for rectal prolapse surgery would therefore be valuable, ideally through the development of multicenter databases with standardized data collection encompassing all surgical approaches used for IRP and ODS recurrences, including stapled rectal resection procedures.

## Supplementary information

Below is the link to the electronic supplementary material.

ESM 1 (DOCX 33.2 KB)

## Data Availability

All data, analytic methods, and study materials used to conduct this research will be made available to any researcher from the corresponding author (CR) upon reasonable request.
